# Engagement intervention versus treatment as usual for young adults with serious mental illness: a randomized pilot trial

**DOI:** 10.1186/s40814-020-00650-w

**Published:** 2020-07-23

**Authors:** Michelle R. Munson, James J. Jaccard, Lionel D. Scott, Sarah C. Narendorf, Kiara L. Moore, Nadia Jenefsky, Andrea Cole, Maryann Davis, Todd Gilmer, Rei Shimizu, Kristin Pleines, Kamilyah Cooper, Aaron H. Rodwin, Lindsay Hylek, Angel Amaro

**Affiliations:** 1 Silver School of Social Work, New York University, 1 Washington Square North, New York, NY 10003 USA; 2 School of Social Work, Georgia State University, Atlanta, Georgia 30302 USA; 3 Graduate College of Social Work, University of Houston, 3511 Cullen Blvd, Houston, TX 77204 USA; 4 New York State Psychiatric Institute, 1051 Riverside Drive, New York, NY 10032 USA; 5 Medical School, Psychiatry, University of Massachusetts, 55 Lake Avenue North, Worcester, MA 01655 USA; 6 Department of Family Medicine and Public Health, University of California San Diego, San Diego, CA 92093 USA; 7 School of Social Work, Columbia University, 1255 Amsterdam Ave, New York, NY 10027 USA

**Keywords:** Young adults, Experimental therapeutics, Serious mental illness, Engagement, Hybrid I Trial

## Abstract

**Background:**

Young adults have elevated rates of mental health disorders, yet they often do not receive consistent care. The challenge of continuing to engage young adults has been pervasive worldwide. Few engagement interventions have been designed for young adults with serious mental illness. *Just Do You* is a theoretically guided engagement intervention. It uses innovative modalities (i.e., technology, expressive arts activities, narrative expression, mentoring) to engage participants in conversations about services and how they work, while simultaneously orienting them to treatment.

**Methods/design:**

This pilot and feasibility study utilizes a hybrid research design, examining feasibility, acceptability, and preliminary impact, alongside implementation. The study combines qualitative methods, a small pilot randomized trial, and a small cost-benefit analysis. Respondents are clinic staff and young adults who have made initial contact with the Personalized Recovery Oriented Services (PROS) program. Quantitative survey data are collected at baseline, 2 weeks (post-intervention), 1 month, and 3 months. The assessments focus on measuring feasibility, acceptability, engagement, and mental health outcomes. Medical record extraction will be used to triangulate self-report data. We will conduct single degree of freedom contrasts to examine whether *Just Do You* leads to improved outcomes relative to Treatment-As-Usual using robust regression for each outcome measure. We will examine whether changes in the proposed mediating variables occur across groups using a similar contrast strategy. In addition, we will use structural equation modeling to examine the contribution of mediators to ultimate outcomes. Finally, we will use constant comparison coding techniques for qualitative analyses.

**Discussion:**

The aim of this study is to examine the feasibility of a young adult engagement meta-intervention through an intensive preliminary pilot trial, learning through collaboration with stakeholders. *Just Do You* has the potential to fill a gap in the service system for young adults with serious mental illnesses, improving the seemingly intractable problem of disengagement. The program uses culturally responsive strategies, is recovery-oriented, and builds upon the best evidence to date. Our efforts align with local and national health care reform efforts embedding people with lived experience.

**Trial registration:**

This trial was registered with ClinicalTrials.gov (Identifier: NCT03423212) on April 18, 2018, as Protocol Record R34 MH111861-01, New York University, as the *Just Do You* Program for Young Adults with Serious Mental Illness

## Introduction

The Institute of Medicine published a report calling on the nation to address gaps in knowledge regarding the health of young adults and to develop health-based interventions, using developmentally and culturally appropriate strategies [1]. The present study addresses the call by empirically testing a theory-driven, promising meta-intervention (an intervention aimed at orienting young adults to treatment interventions) that occurs during the intake process in adult mental health settings. Intake is a critical process that can impact both initial and continued engagement in treatment. It provides an opportunity to welcome new clients, introduce them to social contacts at the agency, and provide them with information about the services provided at the agency. Clients can then make informed decisions about whether or not to invest in their treatment and overall recovery. In the present trial, the intervention under investigation is designed to improve engagement in Personalized Recovery-Oriented Services (PROS), which is a psychiatric day program that incorporates evidence-based treatments, rehabilitation, and social supports for adults with the overall aim of assisting them in achieving their life goals [2]. Our study examines *if* and *how* a brief (2 modules) young adult intervention can improve participants’ engagement in the larger PROS program.

The intervention was designed in collaboration with young adults diagnosed with serious mental illnesses (schizophrenia, bipolar disorder, depression) using participatory research principles [3, 4]. The goal of the intervention is to improve engagement in mental health care among young adults, including enrollment, consistent attendance, and adherence to therapeutic protocols. The intervention provides content and activities that are young adult centered; emphasize motivation, education, and identity; and address common barriers of engagement [4]. Engagement is a complicated construct with varied conceptualizations across studies [5, 6]. In this pilot trial, our team concentrates on engagement among young adults who have made an initial contact with PROS, thereby focusing on common barriers to remaining engaged as opposed to barriers to initial engagement, such as literacy and access. The study defines engagement as “multifaceted” with data collected on the most common behavioral dimensions of treatment engagement, namely, attendance [7], overall level of investment in services when attending the program, and intention to engage in services in the future.

### Application of experimental therapeutics to psychosocial intervention evaluation

In the present trial, we apply experimental therapeutics [8]. This approach, discussed in detail below, frames the research design and statistical approach so that the study can answer the following critical questions: (1) does the intervention impact the primary outcome, namely, engagement; (2) does the intervention impact the hypothesized immediate “targets,” or mechanisms thought to influence engagement (e.g., beliefs about treatment, stigma, mistrust, and hope) [9, 10]; and (3) are the proposed mechanisms thought to be relevant to engagement actually relevant? The study also uses a communication framework for intervention design [11]; that is, our team considered theoretically relevant dimensions of communication when designing the intervention. This included taking into account the source of the communication, the message content, the vehicle of delivery, and the unique characteristics of the targeted audience. This article provides an overview of engagement on the part of marginalized young adults, followed by a discussion of relevant theoretical frameworks. We also outline the aims and methods of the trial, making clear the need for research on young adult engagement.

### Mental health in young adulthood and treatment engagement during and after intake

Mental health conditions can be debilitating and costly [1, 12], especially if they are not treated [13, 14]. Rates of these conditions among young adults are high when compared to older and younger age cohorts [15–17] and even more elevated among young adults who are served by safety-net public systems of care [18]. Mental health conditions often persist from childhood into adulthood [19]. Thus, treatment engagement for young adults is critical and requires attention by researchers with a specific focus on addressing the barriers that exist before young adults’ access treatment, during the intake process, and while young adults are in treatment. Some documented barriers have been identified that are relevant across treatment process periods (e.g., stigma, beliefs about treatment, transportation) while others are more relevant at certain periods than others, for example, lack of knowledge about where to get treatment, which is often a barrier during the pre-intake phase.

Research has shown that if treatment is adhered to consistently, symptoms often decrease and functioning improves [20]. Studies also show that early discontinuation from treatment is associated with an increased likelihood of symptom recurrence [21]. One recent study found that in an urban, low-income sample of young adults hospitalized for serious psychiatric conditions, approximately 50% did not follow-up with treatment following discharge, with African-American and substance using young adults being most vulnerable [22]. Research also highlights that the transition from pediatric to adult healthcare is a particular point of vulnerability for treatment discontinuity and a time when there is an increase in untreated cases [23, 24]. A longitudinal study of child welfare-involved youth reported that while 47.3% used services as adolescents, only 14.3% continued as young adults [25]. Research has also found that the majority of clients who miss their first intake at a community mental health clinic and who report a continued need for treatment did not find services elsewhere for at least another year [26]. Taken together, these studies provide compelling evidence that treatment engagement throughout a mental health episode needs continuous attention and that these lapses in treatment can lead to deterioration in mental health and functional recovery. The engagement intervention that is the focus of this pilot trial seeks to address engagement for young adults who have made an initial contact with treatment, by using innovative young adult informed approaches to reduce drop out and increase engagement in treatment.

### Health disparities among marginalized young adults with serious mental illnesses

Research has documented that young adults who identify as ethnic/racial minorities are more likely to drop out of treatment [27]. While we recognize many young adults are marginalized, our engagement intervention and pilot trial focuses on young adults who largely identify as African-American, Latinx, and bi- or multi-racial. Ethnic/racial minority status has been found to complicate treatment access and engagement [28, 29]. Health disparities among racial and ethnic minority groups are well documented [30, 31]. For mental health services, studies indicate disparities in quality, access, and use [32, 33]. Efforts have been made to address such disparities, but for Black and Latinx individuals, they persist [34]. Studies of treatment dropout have shown that socioeconomic position (being poor) and race (being non-white) are associated with premature termination [35, 36]. This pilot trial addresses this identified area of need by testing the efficacy of a brief innovative meta-intervention designed to improve engagement for marginalized young adults.

### Identifying the immediate targets for change in engagement among marginalized young adults

Our emergent-theoretic research led to a mid-level theory that specifies the underlying mechanisms of engagement in mental health treatment among marginalized young adults with serious mental health conditions [9]. The “young adult framework” [9] integrates the Unified Theory of Behavior (UTB), which is based upon health behavior change and formal decision theory [37] and elements of mental health service use theories, for example, Pescosolido’s Network Episode Model (NEM) [38]. The framework identifies the immediate targets of engagement for marginalized young adults with serious mental illnesses. These targets are hypothesized to directly impact engagement in the current study.

**Fig. 1:**
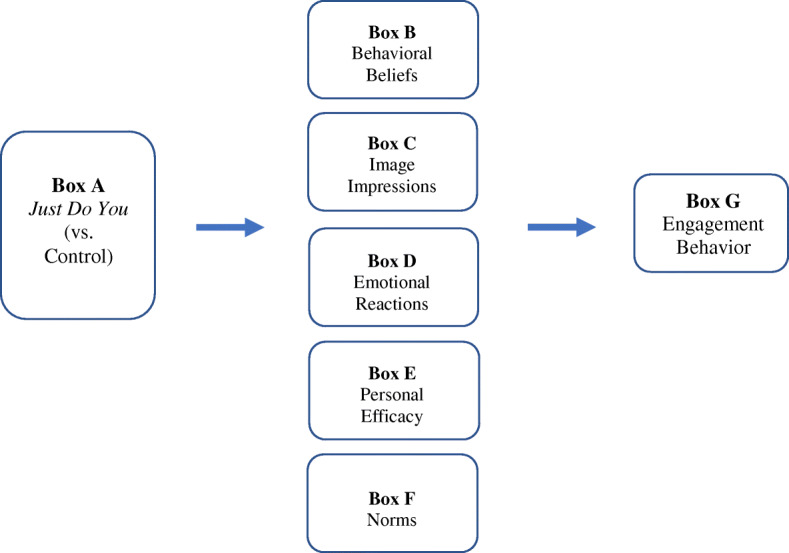
Young adult engagement program: application of experimental therapeutics

In Fig. 1, the left most box (Box A) represents *Just Do You* (the name young adults use for the engagement intervention) versus a control condition, and the right most box represents the primary outcome engagement. Engagement is impacted, in part, by the client’s decision to perform behaviors that constitute engagement (e.g., attend sessions, engage when in treatment sessions). Positive decisions to perform these behaviors are seen as necessary but not sufficient for actual engagement behavior. As such, one purpose of *Just Do You* is to encourage young adults to make strong and informed positive decisions to engage in care and then to translate those decisions into engagement behavior(s). According to the framework, there are five immediate targets that underlie engagement decisions, which are the mechanisms of change (see Boxes B–F below). These are the targets of the engagement intervention.

The five immediate targets of the intervention represent what we call an individual’s “cognitive and affective construction” of what it means to them to engage in professional mental health services. Our framework suggests that to change the outcome of engagement in treatment, an intervention must first change the immediate targets, or underlying mechanisms of engagement [9]. These targets are behavioral beliefs, image management, emotional reactions, social norms, and personal efficacy. Behavioral beliefs (Box B) refer to an individual’s thoughts about the advantages and disadvantages of engaging in professional mental health treatment. For example, a belief that has emerged as salient to marginalized young adults is whether an individual believes that professional treatment can help them with their symptoms [9, 39]. Image management (Box C) refers to the perceived image implications of performing the behavior—the kind of images one thinks they will convey to others if they go to services (e.g., stigma, bravery) and how the behavior fits with one’s own self-concept or identity. Extant research has shown and continues to show that youth and young adults experience stigma that gets in the way of treatment engagement [40–42]. Third, emotional reactions such as fear, hopelessness, and ambivalence may be evoked when thinking about treatment engagement (Box D) [43]. For example, in a study we conducted of marginalized young adults, results revealed that fear was a common emotion experienced when considering engaging in treatment, and interestingly, fear acted as both a motivator and a deterrent to treatment engagement among marginalized young adults [9]. Fourth, personal self-efficacy (Box E), or one’s perceived ability to perform a behavior, has been shown to be associated with actual behavioral performance [44]. Finally, social norms are a young adult’s perception(s) of the level of approval or disapproval of important others in their lives should they decide to engage in treatment (Box F).

It is important to note that the framework for the study does not suggest that young adults carefully consider each of these factors when making decisions. Rather, based on past experiences, cognitions and emotions tied to one or more of these mechanisms can enter working memory in a given situation and influence their behavior. More distal variables, such as personality, values, aspirations, community influence, and social relationships, also can influence decisions, but they usually do so by impacting one or more of these targets. Many studies have found support for these variables in predicting behavior, including mental health service use behavior [9].

Beyond individual-level variables, studies have found that insurance status, access to quality care, transportation, reminder phone calls, child care, and discrimination further complicate the engagement question. Even if an individual wants to get care and is fully invested in treatment engagement, they may face environmental barriers. For example, one study reported that Medicaid lapses were associated with fewer clinic visits [45]. And, studies have illustrated that lack of transportation and child care are barriers to engagement among parents of young children [6]. Our trial recognizes the relevance of these factors.

### How to address the immediate targets: communication strategies

Our pilot trial is designed to address the immediate targets of treatment engagement. Research suggests that it is equally important to make informed decisions about how to communicate with the population of interest to have the best chance at impacting the hypothesized targets of engagement. Based on formative research, our team applied a communication framework for program design [18], including strategies based on perspectives of clinic administrators, staff, and young adults themselves on the most acceptable ways to maintain the attention of young adults and impart change [4, 18].

Our manualized approach focuses on four empirically based communication strategies which emerged in our formative research and that are supported in the larger empirical literature: (1) narrative health communication, (2) co-facilitation and collaboration, (3) expressive arts, and (4) the impact of peers. Each of these strategies is rooted in communication theory which emphasizes one or more of the five facets of health communication which can impact outcomes: (1) the source of the communication; (2) the structure of the message (how information and intervention materials are conveyed); (3) the vehicle through which messages are transmitted; (4) the recipient the message is directed at; and (5) the context in which the communication occurs [18]. Preliminary research, coupled with a literature review [18], informed the communication strategies of the young adult engagement program, which we discuss below.

### Narrative health communication

Narrative health communication provides health-related information through entertainment education, journalism, literature, testimonials, and storytelling [46]. In cancer prevention, narrative expression has been found to decrease client resistance to treatment [47] and increase hope and confidence [48]. In our preliminary research, young adults with mood disorders discussed the desire to share their own story and explain to others their experiences [49]. Thus, the intervention used in this pilot trial employs narrative expression in two ways: (1) celebrity narratives that communicate mental health and treatment engagement messages (e.g., Mary J. Blige, Metta World Peace) and (2) a co-facilitation model that includes a person with lived experience who shares his/her narratives. These narrative tools directly address the immediate targets, or mechanisms, such as challenging stigma and moving from denial to acceptance. Celebrity testimonials and narratives have become commonplace in the era of technology and social media; however, we are not aware of any studies that have empirically tested the use of these narratives to change treatment engagement behaviors among marginalized young adults.

### Co-facilitation and collaboration: licensed clinician and person with lived experience

The communication strategy of using dual providers is important when considering how clients perceive the *source* of the intervention who is communicating health messages. Based on previous research, our hypothesis is that the intervention will have the greatest impact if a licensed clinician delivers it collaboratively with a person with lived experience of mental illness and service use. Research shows that young adults with serious mental illnesses want to learn with others who have similar experiences, those who have “been there” [49]. Communication theory supports this view [18, 50].

Research suggests that there are three source dimensions that are particularly relevant: (1) perceived expertise, (2) perceived trustworthiness, and (3) perceived availability/accessibility [50]. The co-facilitation model considers each of these dimensions. With regard to “perceived expertise,” *Just Do You* recognizes the need for two experts to impact young adults’ beliefs about treatment, specifically a clinical and an experiential expert. Also, placing *Just Do You* in settings where facilitators are present for daily activities increases the perceived availability and accessibility of providers. The clinician provides information on how evidence-based treatments can make a positive impact, while remaining open to discussions on negative past experiences and side effects. The peer provider, referred to as a Recovery Role Model (RRM), facilitates important conversations on the following: (1) acceptance of mental health challenges, (2) acceptance of a need for help, (3) managing stigma, (4) managing life transitions, (5) learning how to advocate for themselves regarding treatment choices, and (6) maintaining hope. The presence of a person with lived experience facilitating the intervention with a clinician also provides a model of a working relationship between someone the young adults likely perceive as “more like me” and a mental health professional they may relate to less. It also highlights the importance of lived experiences as a source of expertise. We hypothesize that co-facilitation will be particularly effective at shaping young adults’ views about mental health services and ultimately improving treatment engagement.

### Communication through creative expression: bringing art and music to mental health treatment

*Just Do You* uses expressive vehicles because young adults report interest in having mental health conversations through creative expression [49]. Further, Malchiodi argues “all expressive therapies focus on encouraging clients to become active participants in the therapeutic process” [51]. The experience of creating activates engagement by energizing individuals, focusing attention, and alleviating emotional dysregulation, thus allowing clients to fully concentrate on their goals. Processing narratives to a repetitive and melodic rhythm has been found to foster neural integration, improve emotion regulation, enhance attunement and a sense of safety, and reduce stress and arousal [52–54]. Van der Kolk also notes that words cannot always integrate the disorganized feelings and thoughts that often exist due to past trauma [55], which many participants have experienced. Art and music go beyond words, offering a language for experiences that may previously have gone unexpressed. Griffiths also discusses how the role of arts in creating opportunities for participation in conversations may be particularly important as it is a protective factor for mental health [56]. Our team designed *Just Do You* to address these potentialities by injecting the pleasurable aspects of art and music into conversations about difficult experiences in the past and possibilities for the future. In a small previous study of *Just Do You*, which focused on acceptability, marginalized young adults reported they liked sharing their creative work [4]. The use of expressive arts is increasing in mental health treatment, and there is a growing body of evidence that supports it as a promising and effective strategy [56–59].

### The power of a relational context

*Just Do You* builds on the core principles of Relational-Cultural Therapy (e.g., authenticity and respect) to create an environment where relationships between interventionists and clients are viewed as central to therapeutic effects [60]. Relational-Cultural theorists propose that through “connection,” which is the outcome of growth-fostering relationships, change happens [60]. Sparks applied this approach and found that being authentic with institutionalized girls was essential to gain trust [61]. *Just Do You* builds on the salience of these important relational characteristics for marginalized young adults. During the period of involvement in the intervention, participants have access to the Recovery Role Model for one-on-one meetings to discuss anything that has arisen from the modules, for example, how to disclose their mental health, how to manage stigma, and/or how to develop goals for their recovery.

### Additional innovation of the young adult engagement intervention *Just Do You*

A systematic review of engagement interventions suggested there are few engagement programs focused on young adults [6]. As well, few engagement interventions targeting children and youth focus on the cognitive and affective processes that underlie engagement behaviors (see [62] for an example). Instead, most engagement interventions rely on environmental/contextual strategies (e.g., reminder calls, in home visits) and family interventions [6, 43]. Indeed, both are important. Our intervention—the first of its kind—offers a “meta intervention” focusing on moving beyond environmental barriers to examine how young adults think and feel about treatment, as they are increasingly making their own treatment decisions. Modules validate past treatment experiences that were not helpful, while discussing approaches that may be helpful now. Providers process these experiences, while acknowledging environmental barriers. Young adults are making decisions about treatment, decisions that involve their emotions and thoughts.

Most efforts to improve the mental health among transition-age youth focus on extending the age of programs in the children’s system [63]. Although this strategy has had some success in maintaining transition-age youth in treatment, the success has been modest. Also, some young adults do not make initial contact with treatment until they are adults. The field needs additional strategies that strengthen engagement efforts when young adults make contact in the adult system. Further, large numbers of youth do not remain in services past age 18, in part, because they have more autonomy in decision-making [23], and, in part, because of negative past treatment experiences [11]. There is some research on treatment engagement in the children’s systems, yet, there is little research on engagement in the adult system [64].

### Summary and pilot trial research questions

This pilot trial tests, in a preliminary way, a program created with marginalized young adults to address barriers to treatment engagement among those experiencing social and economic marginalization. Young adulthood is a period when individuals begin to assert developmentally appropriate autonomy in their health care decisions, decisions that can impact their lives for years. The goal is to bring young adults “on board” with their treatment and overall recovery. The reason for the pilot trial is to refine study protocols, examine acceptability, and examine preliminary impact on mediating targets of engagement and engagement. The research questions are (1) is it feasible to provide the young adult engagement program at PROS programs?; (2) is the program acceptable to stakeholders?; and (3) does the intervention show a signal of positive impact on mediators and engagement, and if so, gain insight into how the impact is made?

## Methods

### Overview

The study uses a parallel design with two groups (i.e., treatment versus control) with random assignment to condition and a 1:1 allocation ratio. Random assignment is accomplished with computer-generated random numbers. The study Principal Investigator provides the number sequences to the Project Director who compiles the study packets in ways that assure randomization to condition. Clinical and research staff nor young adult participants are blind to the study condition. The study will obtain four assessments: (baseline, a 2-week post-test, a 4-week follow-up, and a 3-month follow up). The 4-week follow-up is used, in part, to reduce attrition, as previous research shows that maintaining young adults in longitudinal research is difficult. The effects of the intervention on treatment engagement outcomes, such as attendance, intention, and overall level of investment, will be assessed. The CONSORT checklist delineating all elements of the study protocol and where they are discussed is in the journal on-line supplement. The SPIRIT figure appears in Table 1.

**Table 1:**
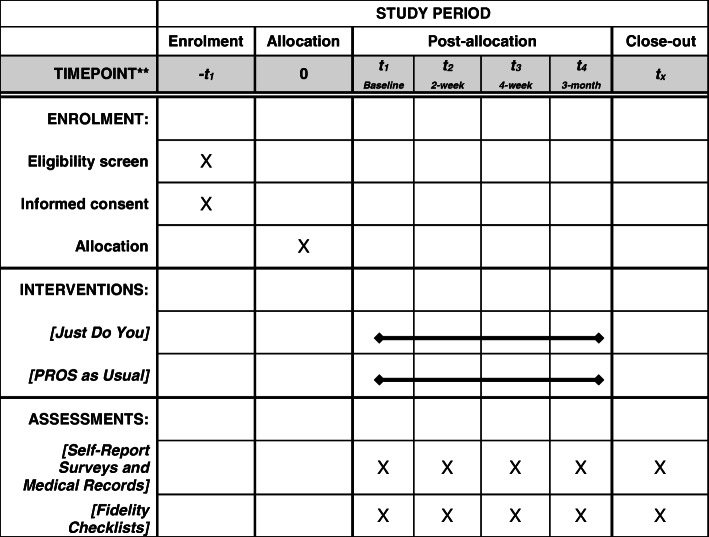
Randomized pilot trial of an engagement intervention for young adults

Our project aims to recruit 195 marginalized young adults into the study, with 97 in the treatment condition and 98 in the control condition. The control condition receives Treatment-As-Usual (TAU), which in this case means beginning PROS as soon as enrolled without a standardized orientation to the program and services. We use a within-site randomization design. We do not expect contamination, but we will directly assess both participant and provider contamination through assessments with both treatment and control staff and study participants.

The project consists of three phases. During Phase 1, research offices are prepared and staffed, protocols are refined and modified with stakeholder input, and approvals of all human subject protocols are obtained from the requisite organizations (e.g., university, agency, private). The team also hired and trained project research staff and conducted all trainings for interventionists. In Phase 2, primary data collection takes place for the trial. In Phase 3, focus turns to completion of analyses and dissemination.

### Sample

Participants are recruited from four sites in one urban city that provides personalized recovery-oriented services (PROS). PROS are outpatient programs that provide recovery-oriented services, including evidence-based treatments (e.g., integrated dual disorders treatment, psychoeducation), rehabilitation services, and support to adults with serious mental illness [2]. The inclusion criteria for the trial are as follows: (1) adults between the ages of 18 and 34, (2) living with a serious mental illness (i.e., mood, anxiety, schizophrenia spectrum), (3) are in the intake process or enrolled at PROS, and (4) were formerly involved with public safety net systems of care. Exclusion criteria are (1) cognitive impairments (i.e., young adults who cannot understand the consent process) and (2) non-English speaking young adults (we will include individuals whose primary language is not English but are able to comprehend and speak English). Respondents are informed of the study during the intake process by a project staff member. At PROS, intake is generally a 2- to 4-week process which includes assessments and general conversation about the program. If interested in participating in the study, they are invited to an introductory information session with a research staff member who explains the study protocols and provide time for potential participants to ask questions. Those who agree to be in the study begin the informed consent process with a research staff member.

Typically, the participants in the experimental condition begin the intervention within a week of their initiation to the project, and this occurs within a window of 2 to 4 weeks of their arrival at PROS. We expect no selection effects at recruitment but are testing for them by comparing people who agree versus a subset of those who refuse to be in the study. Respondents are paid $20 for each completed assessment. The program is designed for new clients at PROS, but when the pilot commenced, we changed the method to include both new and already enrolled young adults to increase recruitment. We will compare the two groups on study outcomes. Clinic administrators reported that at any given point in time, there were between 50 to 75 young adults attending their PROS programs. They report enrolling, on average, 5 new young adults a month who meet eligibility criteria. With the service engagement intervention budgeted to run for 20 months, it is expected that we should be able to recruit 195 young adults, given an estimated 30% refusal rate.

### Sample attrition and retention

The team has extensive experience and success tracking difficult to reach populations. We use standard methods for tracking difficult to reach populations, such as on-going reminder calls. In addition, contact information for the young adults and people who can reach them are obtained and revisited at each assessment. A computerized tracking system from our prior research will be used.

### Setting and location: the partnering clinics

Our collaborating partner(s) are two large behavioral health organizations. Each of these partners provides PROS to adults in low-resourced communities, and each has discussed with the research team the challenges in maintaining young adults in their programs. The partnering programs are licensed, comprehensive programs for adults with serious mental illnesses that integrate evidence-based treatment and numerous types of support and rehabilitation services.

### Randomization

After being introduced to the project, and consenting to involvement, the participant receives a study packet, which includes a condition card determined earlier by the computer-generated random numbers. The condition cards are placed in the packets and used by staff to implement the random assignment protocol.

### Description of the *Just Do You* intervention program

The *Just Do You* intervention is a two module intervention designed to improve the level of engagement in mental health care among young adults. The two 90-min modules are provided by a licensed clinician and a person with lived experience who has a mental health diagnosis and uses professional treatment. Modules use young adult centered activities that focus on motivation, education, identity, and addressing barriers of engagement. There is an intervention team at each site. Each of the sessions is designed with the following features: a “relational” environment, process-oriented strategies, and a curriculum that centers on the empirically based underlying mechanisms of engagement. See Table 2 for a description of module content and activities. Both the Just Do You group and the Treatment-As-Usual group receive the same Personalized Recovery Oriented Services (PROS).

**Table 2:** *Just Do You* two-session protocol

Session	Activities	Immediate targets addressed
Session 1	Welcome/de-bunking myths/developing narratives 1. Welcome; group guidelines, purpose 2. Discuss SAMHSA recovery principles 3. Narrative of role model (experiences with the system, moving from distrust to some trust) 4. Video of celebrity service user (discussion) 5. Recovery goals (and role of services in goals)	Image impressions (i.e., stigma) Emotions (i.e., hope) Behavioral beliefs (i.e., trust, understanding services/literacy)
Session 2	Literacy/knowledge/validating past/instilling hope 1. What are services and how can they help? (psychoeducation) 2. Visual art exercise—cause of SMI and validation of past experiences (psychoeducation) 3. Maintaining my Medicaid insurance 4. Discussion of systemic barriers	Behavioral beliefs (i.e., services can help) Efficacy (i.e., advocacy) Knowledge/environmental barriers (i.e., cause) Emotions (i.e., hope, fear, ambivalence)

### Treatment-As-Usual condition

Treatment-As-Usual participants receive the regular intake process and the PROS protocol of services for adults with serious mental illness. The intake process consists of a series of meetings and assessments with the intake staff at the site. PROS treatment is delivered through psychoeducation, skills based, and therapeutic groups, along with pharmacology. Staffing includes a multi-disciplinary team of social workers, peer specialists, psychiatric nurses, and mental health counselors. Possible goals for PROS participants are to improve functioning, reduce inpatient hospitalization, reduce emergency services, reduce contact with the criminal justice system, increase employment, attain higher levels of education, and secure housing. PROS are currently used throughout the state where the trial is taking place.

### Qualifications, hiring, and training of project staff

The clinician providers are employees working at the partnering sites. They were chosen based on their combined expertise on young adults and expressive arts. They are licensed and trained to provide *Just Do You* based on the intervention protocols. The peer providers in two cases applied to an advertisement for the role and were interviewed by project staff. In the other two cases, they were already certified peer support providers at the PROS sites. We hired peer providers who are active in their recovery and are interested and able to speak about their experiences and who were approximately a decade older than participants. Our training requirement was for peer providers, at a minimum, to complete the online training modules which are part of the process of becoming a certified peer specialist in our state and complete our intervention training. We did not require them to complete certification, which in New York requires fees, clinical documentation, and letters of reference. Once all sites had peer providers, our staff trained them on the manualized intervention protocols. Also, the project team meets regularly to provide support and process how the provision of *Just Do You* is going at PROS programs.

All staff are certified by the partnering university’s Human Subjects program. Staff are trained on the informed consent process, Good Clinical Practices, all safety and reporting protocols, and assessments. The clinic staff are trained by the Principal Investigator on the *Just Do You* manualized approach and all reporting procedures for safety, risk, and clinical crises. Our team uses an initial training protocol, and we offer continued training periodically, including any time there is staff turnover.

### Process evaluation: supervision, treatment fidelity, and monitoring

To determine the extent to which the program is delivered as intended, a number of criteria are examined. Interventionists use session checklists to self-monitor the content delivered. Treatment fidelity is monitored by staff raters who observe select sessions and rate the content delivered using checklists. Weekly supervisory meetings are held to review cases. Interventionists keep a log that records attendance at groups, level of participation in group activities, and issues that arise. Lastly, feedback is given to the project team on issues that come up, and they are dealt with in a timely fashion by project supervisors. Young adults are also asked to evaluate the program in terms of satisfaction on the content and process using focus groups and individual interviews.

### Measures

All of the measures used in this project have been evaluated in our pilot research, and all have been found to have good psychometric properties. Table 3 includes key constructs we are assessing as part of the pilot randomized trial and all study measures. Data for the pilot trial will be made available in the NIH National Data Archive.

**Table 3:** Measures utilized in the young adult engagement trial

Outcomes/Constructs Study vs. Validated Scales (S vs. V)	Dimensions & Sample Item	Items & Response Options	Assessment(s) Measured
Primary Outcome Engagement (S and V) S	Attendance at Engagement Intervention Modules	Attendance (Provider Report)	Tracked weekly
S	Intention to Use: “I intend to go to my appointment … at ____?”	5 point Agree/Disagree Scale (Self-Report) [65]	Baseline, 2-wks, 4-wks, 3-mos
S	Adherence to Appointments: “How often have you taken your medication as prescribed?	4 point scale from All of the time to Never (Self-Report)	Baseline, 2-wks, 4-wks, 3-mos
V The Yatchmenoff Client Engagement Scale [66]	Engagement: “I’m not just going through the motions. I’m really involved in working with staff.” Engagement Indicators from Electronic Records	8-items, 5-point agree/disagree scale (Self-Report) [66]	Baseline, 2-wks, 4-wks, 3-mos
S	Number of weeks involved at ____ Number of distinct days present at ____ Number of doctor appointments attended Number of no shows Number of services received	Protocol with Electronic Health Records at Partnering Clinics (Medical Records); Corroborated with self-report data on attendance at ___	Following Data Collection at each Clinic Site
Mediating Outcomes Behavioral Beliefs			
V Fishbein & Ajzen Items	Advantages/Disadvantages of Services: “Continuing my treatment will provide me with non-judgmental support”	Adapted from Fishbein & Ajzen, 2010 [65]	Baseline, 2-wks, 4-wks, 3-mos
S	Mental Health Trust/Mistrust: “I trust that the mental health care staff here are sincerely working to improve my mental health”	4 items, Likert scale, 1 (Definitely False) to 8 (Definitely True)	Baseline, 2-wks, 4-wks, 3-mos
Image Impressions V Fishbein & Ajzen Items	Image Management: “If others who are important to me found out that I follow up with my treatment, I would be seen by them as brave.”	5-point A/D scale [65]	Baseline, 2-wks, 4-wks, 3-mos
S	Attitudes Toward Seeking Mental Health Services [67]	4-item Likert scale; Disagree/Agree	Baseline, 2-wks, 4-wks, 3-mos
Emotions V Fishbein & Ajzen Items	Emotional Reactions: “When I think about the idea of continuing my medication it makes me anxious.”	5-point A/D scale [65]	Baseline, 2-wks, 4-wks, 3-mos
V Snyder’s Hope Scale	Hope: “I energetically pursue my goals.” [68]	12 items, 8 point Likert scale, Definitely False/Definitely True	Baseline, 2-wks, 4-wks, 3-mos
S	Mental Health Hope: “I am more hopeful about my future because I have found ways to manage my mental health condition”	4 items, Likert scale, 1 (Definitely False) to 8 (Definitely True)	Baseline, 2-wks, 4-wks, 3-mos
Social Norms V Fishbein & Ajzen Items	Social Norms: “How would your mother feel about you continuing your treatment for your difficulties (‘issues’) at this time in your life?”	8-items, 6 point approval/ disapproval scale [65]	Baseline, 2-wks, 4-wks, 3-mos
Self-Efficacy V Fishbein & Ajzen Items	Perceived Behavioral Control (4-items from autonomy factor and 4-items from capacity factor)	7-items, 5 point Likert scale [65]	Baseline, 2-wks, 4-wks, 3-mos
Secondary Outcomes			
Recovery V	Recovery Assessment Scale [69]	20-items, 5 point Likert Scale	Baseline, 2-wks, 4-wks, 3-mos
Symptoms V	Colorado Symptom Inventory [70]	10-items, 5-point Likert Scale	Baseline, 2-wks, 4-wks, 3-mos
Fidelity Outcomes S	Clinician Checklists: Session Content Checklists	6-items per session, piloted, Likert scale 1 (Not at all) to 4 (Completely) executed	After every Module
S	Researcher Observations: Checklists and notes on observations of the modules	6-items per session, piloted, Likert scale 1 (Not at all) to 4 (Completely( executed	Selected Sessions

### Strategic developmental science: learning opportunities for future trials

The study uses three activities to provide perspectives on the eventual uptake and sustainability of the young adult engagement intervention: (1) decision-maker meetings, (2) cost-benefit pilot work, and (3) academic-community partnerships. First, we convene periodic meetings of decision-makers at the state and local levels to discuss uptake of the intervention designed to improve engagement in PROS programs. These meetings are important for process and include time for staff to problem solve about difficulties with recruitment, acceptability, and/or feasibility of the study. Second, we engage in research to estimate intervention costs to provide the foundation for a full cost-benefit component in a future trial. Specifically, the incremental costs of the intervention will be estimated from the health system perspective. The costs will be estimated using a combination of process mapping and time-driven, activity-based costing [71, 72]. Process mapping elucidates each element involved in the implementation of the program, including clinic resources and staff that are involved in the integration of the intervention into the clinic workflow. Time-driven, activity-based costing is assigned based on levels of effort and costs associated with each activity identified in the process mapping. Approximate estimates of costs will be made using standard accounting methods and administrative budgets. We are identifying ways of quantifying the benefits of the intervention as well.

### Data analysis

#### General Issues

Missing data will result from either non-response to selected items within a scale or attrition. Missing data will be addressed using either FIML or Markov Chain Monte Carlo multiple imputation strategies [73]. If preliminary analyses suggest a systematic pattern of data that violates missing at randomness, strategies that represent the missingness will be incorporated into the modeling process [73]. The present study focuses on preliminary theory tests and treatment efficacy, so intent-to-treat (ITT) analyses are premature. Nevertheless, we will explore both ITT and per protocol analytic perspectives in the data. We will document non-normality, variance heterogeneity, specification error, outlier effects, and where possible, biasing effects of measurement error in all analyses. Robust methods of analysis (e.g., Huber-White robust standard errors, bootstrapping) will be used, as appropriate [74]. We will make analytic adjustments for cluster effects due to clinics and clinician if intraclass correlations suggest the need for such adjustment [75]. For all multi-item measures, we will evaluate composite reliabilities and factor structures to ensure they behave in a way one would expect based on their psychometric histories. We will routinely examine intercorrelations of variables within a category and, coupled with substantive criteria and the results of exploratory or confirmatory factor analyses, make decisions about combining indices, introducing latent constructs into the analysis, or treating measures in a way that respects their unique variance. The use of power demeaning control for family-wise error rates is not appropriate in feasibility studies like this, so we will not use them, but we will be sensitive to the relevant issues surrounding family-wise error.

#### Specific analyses

Evaluation of the overall effects of the intervention on a given mediator or outcome will be tested by single degree of freedom contrasts comparing mean scores between groups at a given time point. More complex mediational analyses can be pursued using structural equation modeling (SEM) as guided by Fig. 1 but are too sample size demanding for a pilot test. However, we can obtain preliminary estimates of path coefficients linking the treatment condition to the mediators and, in turn, estimate the paths linking the mediators to the more distal outcome of engagement at different time points using regression analyses in a limited information estimation framework. In essence, we will use the framework of a randomized explanatory design (RED) for treatment evaluation [76]. Traditional outcome only studies focus only on the link between the treatment (the left most box in Fig. 1) and a distal outcome (the right most box). By contrast, our analysis will analyze individual links in mediational chains between these variables. For example, if we find no association between the treatment and a given mediator (e.g., behavioral beliefs), this will tell us that the engagement program failed to change a targeted mediator and needs to be revisited accordingly. If a path between a mediator and a key outcome is statistically non-significant, then this indicates that change in the presumptive mediator is not associated with changes in engagement, which suggests we can streamline the program by eliminating that mediator. Overall, the use of a RED provides important feedback about features of the young adult engagement program that seem to work well or that need to be improved.

The analyses of program effects on mediators will focus on the immediate posttest. The linkages between the mediators and our primary outcome (engagement) require linking such changes to engagement indicators at the 3-month posttest. We hypothesize that there will be less decay in effects, if any, from the 4-week to 3-month follow-up in the experimental condition as compared to the control groups, because *Just Do You* should result in greater buy-in to treatment as time goes on.

#### Sample size

This is a feasibility study designed to show design viability and treatment promise. Our outcomes generally will be mean scores reflecting the mediators or degree of compliance-engagement. For single degree of freedom contrasts comparing two groups on means, the required sample size to have power of 0.80 to detect a Cohen effect size of *d* = 0.50 (a medium effect size) is 65 per group, which is our targeted sample size. Using a limited information estimation approach, if a given linear equation implied by Fig. 1 has up to 5 predictors with a squared multiple correlation of 0.30 (which is reasonable given our use of baseline covariates) and a two-tailed alpha of 0.05, a sample size of 125 yields approximate power to detect a regression coefficient that accounts for 5% unique explained variance with power of 0.80. We also will explore the use of specialized SEM methods for small sample sizes, such as Swain-based estimation [75].

#### Qualitative analysis

Qualitative methods will be used three times: (1) refinement of the *Just Do You* protocol based on acceptability and satisfaction data from stakeholders, (2) contamination interviews, and (3) preliminary cost-benefit data. Interviews and meetings for these activities will produce large volumes of field notes and additional documents. The data will be analyzed using constant comparison to develop overarching core themes that will help build understanding for later trials (thematic analysis) [77]. Analysis will proceed in two steps. In step 1, emergent themes are identified through a standard coding process within and between transcripts, and field notes, that leads to the development of a project codebook. Step 2 involves systematic coding using a well-defined thematic codebook. Trained coders will conduct all coding. These analyses are not intended to develop theory, but rather build understanding regarding important next steps for the program of research aimed at improving engagement among marginalized young adults with serious mental illness, and ultimately improving their mental health and well-being. Results will also assist in modifying protocols and procedures for *Just Do You*.

### Summary and future directions

The present study builds on previous research to design an evidence-informed engagement intervention for marginalized young adults. The project provides the opportunity to move the field of mental health services research in the following ways: (1) empirically identify potential mechanisms underlying engagement outcomes, thereby contributing to experimental therapeutics; (2) decrease the number of marginalized young adults with untreated mental illness; (3) shift the negative “cognitive construct” among marginalized young adults regarding how professional services can help; and ultimately, (4) improve young adult mental health and recovery outcomes.

### Ethics and dissemination

The protocol for all research aims has been reviewed and approved by the University Committee on Activities Involving Human Subjects as of December 19, 2017 (IRB-FY2017-1002). Consent to enroll in the study will be processed and obtained by the PI or project research staff who have been trained on activities involving human subjects. Further, BRANY, a private review board reviewed and approved the clinic staff’s involvement in providing the intervention as part of the trials.

## Discussion

Pilot trials are not without limitations. The intervention in the current study could be at risk for variation in optimal implementation because it is being provided at four different PROS clinics with four different provider teams and larger organizational systems and structures. We have built in procedures to reduce this possibility, such as a manualized training process, and fidelity checklists, but it remains a possibility we will examine closely. Further, there is a possibility for contamination bias, if young adults in different conditions share what they are learning. As reported above, we are collecting data to examine this possibility. The study will have limited generalizability; however, if the pilot trial shows promise, the intervention protocol can be scaled up to be evaluated in multiple populations.

Given the ongoing challenges of engaging marginalized young adults in mental health services and the pervasive outcomes that untreated mental illness can have on individuals, families, and society, an intervention expressly designed to address continued engagement in mental health care is needed. This research has the potential to contribute to the understanding of how to decrease the number of young adults with unmet mental health needs by focusing on the empirically identified barriers to continued engagement. This can be accomplished through a collaborative partnership with professionals who work in the adult mental health system, young adult mental health professionals, and young adults’ living with serious mental health conditions themselves. The testing of this intervention is timely given the changes in the public and private insurance system(s) through the Affordable Care Act and State Medicaid Redesign, both of which are likely to result in more young adults having access to health insurance and therefore mental health services

### Trial status

All hiring and training of experimental site personnel and research staff have been completed. We have recruited and enrolled 100 participants for Aim 2 (the Trial) of the discussed study protocol.

## Data Availability

Not applicable

## Abbreviations

RCT: Randomized clinical trial
RED: Randomized explanatory design
JDY: *Just Do You*
TAU: Treatment-As-Usual
PROS: Personalized Recovery Oriented Services
SEM: Structural equation modeling
RRM: Recovery Role Model

## References

[1] Institute of Medicine and National Research Council. Investing in the Health and Well-Being of Young Adults. Washington, DC: The National Academies Press. 2015. 10.17226/18869. Accessed on 9 August, 2019. 25855847

[2] McNabb, Blizzard, & Merrill. The PROS Program Manual, New York State Office of Mental Health. 2017. Accessed September 3, 2018.

[3] Whyte WF (1991). Participatory Action Research. Sage focus editions, Vol. 123.

[4] Munson MR, Cole A, Jaccard J, Kranke D, Farkas K, Frese FJ (2016). An Engagement Intervention for Young Adults with Serious Mental Health Conditions. J Behav Health Serv Res.

[5] Gibbons MBC, Gallop R, Thompson D, Gaines A, Rieger A, Crits-Christoph P (2019). Predictors of treatment attendance in cognitive and dynamic therapies for major depressive disorder delivered in a community mental health setting. J Consult Clinical Psych.

[6] Kim H, Munson MR, McKay MM (2012). Engagement in mental health treatment among adolescents and young adults: A systematic review. Child Adolesc Soc Work.

[7] Becker KD, Boustani M, Gellatly R, Chorpita BF (2018). Forty Years of Engagement Research in Children’s Mental Health Services: Multidimensional Measurement and Practice Elements. J Clin Child Adolesc Psych.

[8] Raghavan R, Munson MR, Le C. Toward an Experimental Therapeutics Approach in Human Services Research. Psychiatr Serv. 2019:appips201800577. 10.1176/appi.ps.201800577 31500543

[9] Munson MR, Jaccard J, Smalling SE, Kim H, Werner JJ, Scott LD (2012). Static, dynamic, integrated, and contextualized: a framework for understanding mental health service utilization among young adults. Soc Sci Med.

[10] Dixon LB, Holoshitz Y, Nossel I (2017). Treatment engagement of individuals experiencing mental illness: review and update. World Psychiatry.

[11] Munson MR, Jaccard J (2018). Mental Health Service Use Among Young Adults: A Communication Framework for Program Development. Admin Pol Ment Health.

[12] Luciano A, Meara E (2014). Employment status of people with mental illness: National survey data from 2009 and 2010. Psychiatr Serv.

[13] Government Accountability Office. Behavioral Health: Research on Costs of Untreated Conditions is Limited (Report GAO-19-274). 2019; Retrieved from gao.gov on 1 October, 2019.

[14] Murray CJ, Lopez AD (1996). The global burden of disease and injury series: A comprehensive assessment of mortality and disability from diseases, injuries, and risk factors in 1990 and projected to 2020.

[15] Kessler RC, Birnbaum H, Bromet E, Hwang I, Sampson N, Shahly V (2010). Age differences in major depression: results from the national comorbidity survey replication (NCS-R). Psychol Med.

[16] Twenge JM, Cooper AB, Joiner TE, Duffy ME, Binau SG (2019). Age, period, and cohort trends in mood disorder indicators and suicide-related outcomes in a nationally representative dataset, 2005-2017. J Abnorm Psychol.

[17] Blanco C, Okuda M, Wright C, Hasin DS, Grant BF, Liu SM, Olfson M (2008). Mental health of college students and their non-college-attending peers: Results from the National Epidemiologic Study on Alcohol and Related Conditions. Arch Gen Psychiatry.

[18] Center for Behavioral Health Statistics and Quality. Behavioral health trends in the United States: Results from the 2014 National Survey on Drug Use and Health (HHS Publication No. SMA 15-4927, NSDUH Series H-50). 2015. Retrieved from http://www.samhsa.gov/data/.

[19] Kim-Cohen J, Caspi A, Moffitt T, Harrington H, Milne B, Poulton R (2003). Prior juvenile diagnoses in adults with mental disorder: developmental follow-back of a prospective-longitudinal cohort. Arch Gen Psychiatry.

[20] Butler AC, Chapman JE, Forman EM, Beck AT (2006). The empirical status of cognitive-behavioral therapy: A review of meta-analyses. Clin Psychol Rev.

[21] Melfi CA, Chawla AJ, Crogham TW, Hanna MP, Kennedy S, Sredl K (1998). The effects of adherence to antidepressant treatment guidelines on relapse and recurrence of depression. Arch Gen Psychiatry.

[22] Marino L, Wissow L, Davis M, Abrams MT, Dixon LB, Slade EP (2015). Predictors of outpatient mental health clinic follow-up after hospitalization among Medicaid-enrolled young adults. Early Interv in Psychiatry.

[23] McMillen JC, Raghavan R (2009). Pediatric to adult mental health service use of young people leaving the foster care system. J Adolesc Health.

[24] Copeland WE, Shanahan L, Davis M, Burns B, Angold A, Costello EJ (2015). Untreated Psychiatric Cases increase during the Transition to Adulthood. Psychiatr Serv.

[25] Ringeisen H, Casanueva CE, Urato M, Stambaugh LF (2009). Mental health service use during the transition to adulthood for adolescents reported to the child welfare system. Psychiatr Serv.

[26] Peters FPML, Bayer J (1999). “No-show” for initial screening at a community mental health centre: Rate, reasons, and further help-seeking. Soc Psychiatry Psychiatr Epidemiol.

[27] McFarland BR, Klein DN (2005). Mental health service use by patients with dysthymic disorder: Treatment use and dropout in a 7 1/2-year naturalistic follow up. Compr Psychiatry.

[28] Turner HA, Finkelhor D, Ormrod R (2006). The effect of lifetime victimization on the mental health of children and adolescents. Soc Sci Med.

[29] Turner HA, Butler MJ (2003). Direct and indirect effects of childhood adversity on depressive symptoms in young adults. J Youth and Adolesc.

[30] Dobalian A, Rivers PA (2008). Racial and ethnic disparities in the use of mental health services. J Behav Health Serv Res.

[31] U.S. Department of Health and Human Services. Mental Health: Culture, Race, and Ethnicity—A Supplement to Mental Health: A Report of the Surgeon General. Rockville, MD: U.S. Department of Health and Human Services, Substance Abuse and Mental Health Services Administration, Center for Mental Health Services. 2001. 20669516

[32] Alexandre PK, Martins SS, Richard P (2009). Disparities in mental health care for past-year major depressive episodes among Caucasian and Hispanic youths. Psychiatr Serv.

[33] Harris KM, Edlund MJ, Larson S (2005). Racial and ethnic differences in the mental health problems and use of mental health care. Med Care.

[34] Brady J, Ho K, Kelley E, Clancy CM (2007). AHRQs national healthcare quality and disparities reports: An ever-expanding road map for improvement. Health Serv Res.

[35] Miranda J, Green BL, Krupnick JL, Chung J, Siddique J, Belin T, Revicki D (2006). One-year outcomes of a randomized clinical trial treating depression in low-income minority women. J Consult Clin Psychol.

[36] Barrett MS, Chua W, Crits-Christoph P, Gibbons MB, Casiano D, Thompson D (2008). Early withdrawal from mental health treatment: Implications for psychotherapy practice. Psychotherapy..

[37] Jaccard J, Dodge T, & Dittus P. Parent-adolescent communication about sex and birth control: a conceptual framework. In S. Feldman, & D. A. Rosenthal (Eds.), Talking sexuality: Parent/adolescent communication. In Damon, W. (Ed.), New directions in child and adolescent development, San Francisco: Jossey-Bass, 2002. 10.1002/cd.48 14964942

[38] Pescosolido B. Organizing the sociological landscape for the next decades of health and health care research: the network episode model III-R as cartographic subfield guide. In BA Pescosolido, JK Martin, JD. McLeod, & A Rogers (Eds.), Handbook of the sociology of health, illness, and healing (pp. 39e66). New York: Springer, 2011.

[39] Mendenhall AN, Fristad MA, Early TJ (2009). Factors influencing service utilization and mood symptom severity in children with mood disorders: Effects of multifamily psychoeducation groups. J Consult Clin Psychol.

[40] Kranke DA, Floersch J, Kranke BO, Munson MR (2011). A qualitative investigation of self-stigma among adolescents taking psychiatric medication. Psychiatr Serv.

[41] Golberstein E, Eisenberg D, Gollust SE (2008). Perceived stigma and mental health care seeking. Psychiatr Serv.

[42] Downs MF, Eisenberg D (2012). Help seeking and treatment use among suicidal college students. J Amer Coll Health.

[43] Moore KL (2018). Mental health service engagement among underserved minority adolescents and young adults: a systematic review. J Racial Ethn Health Disparities.

[44] Bandura A (1977). Social Learning Theory.

[45] Slade EP, Wissow LS, Davis M, Abrams MT, Dixon LB (2014). Medicaid Lapses and Low Income Young Adults' Receipt of Outpatient Mental Health Care after an Inpatient Stay. Psychiatr Serv.

[46] Kreuter MW, Green MC, Cappella JN, Slater MD, Wise ME, Storey D, Clark EM, O’Keefe DJ, Erwin DO, Holmes K, Hinyard LJ, Houston T, Woolley S (2007). Narrative communication in cancer prevention and control: A framework to guide research and application. Ann Behav Med.

[47] Dal Cin S, Zanna MP, Fong GT (2004). Narrative persuasion and overcoming resistance. In E. S. Knowles & J. Linn (Eds.), Resistance and persuasion (pp. 275-191).

[48] Chelf J, Deshler A, Hillman S, Durazo-Arvizo R (2000). Storytelling: A strategy for living and coping with cancer. Cancer Nurs.

[49] Munson MR, Lox J (2012). Clinical social work practice with former system youth with mental health needs: Perspectives of those in need. Clin Soc Work J.

[50] Jaccard J (2009). Unlocking the contraceptive conundrum: Reducing unintended pregnancies in emergent adulthood.

[51] Malchiodi C (2005). Expressive Therapies.

[52] Siegel DJ (2010). The mindful therapist: A clinician’s guide to mindsight and neural integration.

[53] Lin S, Yang P, Lai C, Su Y, Yeh Y, Huang M, & Chen C. Mental health implications of music: Insight from neuroscientific and clinical studies. Harv Rev of Psych. 2011;19(1), 34–46. 2011. doi:10.3109/10673229.2011.549769. 10.3109/10673229.2011.549769 21250895

[54] Chanda ML, Levitin DJ (2013). The neurochemistry of music. Trends Cogn Sci.

[55] Sykes Wylie, M. The Limits of Talk: Bessel van der Kolk wants to transform the treatment of trauma. Psychotherapy Networker, 2012.

[56] Griffiths S (2003). Arts and creativity: a mental health promotion tool for young African and Caribbean men. Mental Health Review.

[57] Gillam T (2013). Creativity and Mental Health Care. Ment Health Pract.

[58] Fancourt, D., & Finn, S. What is the evidence on the role of the arts in improving health and well-being? A scoping review. 2019; Retrieved from Copenhagen: World Health Organization Regional Office for Europe. 32091683

[59] Gillam T (2018). Enhancing public mental health and wellbeing through creative arts participation. J of Pub Mental Health.

[60] Miller JB, Stiver IP (1997). The healing connection: How women form relationships in therapy and in life.

[61] Sparks E, Walker M, Rosen WB (2004). Relational experiences of delinquent girls: A case study. How connections heal: Stories from relational-cultural therapy.

[62] Mistler L, Sheidow AJ, Davis M (2016). Trans-Diagnostic Motivational Enhancement Therapy to Reduce Treatment Attrition: Use in Emerging Adults. Cogn Behav Pract.

[63] Munson MR. “Multi-Component Engagement Program -- Considerations for Service Provision to Young Adults Early in Their Recovery,” Invited Webinar, iSPARC Transitions to Adulthood Center for Research, University of Massachusetts Medical School. 2019.

[64] Governmental Accountability Office. Young adults with serious mental illness: Some states and federal agencies are taking steps to address their transition challenges.#GAO-08-678. 2008.

[65] Fishbein and Ajzen (2010). Predicting and Changing Behavior: The Reasoned Action Approach.

[66] Yatchmenoff DK (2005). Measuring client engagement from the client’s perspective in nonvoluntary child protective services. Res Soc Work Pract.

[67] MacKenzie CS, Knox VJ, Gekoski WL, Macaulay HL (2004). An adaption and extension of the attitudes towards seeking professional psychological help scale. J Appl Soc Psychol.

[68] Snyder CR, Harris C, Anderson JR, Holleran SA, Irving LM, Sigmon ST (1991). The will and the ways: Development and validation of an individual-differences measure of hope. J Pers Soc Psychol.

[69] Corrigan PW, Salzer M, Ralph RO, Sangster Y, Keck L (2004). Examining the factor structure of the Recovery Assessment Scale. Schizophr Bull.

[70] Boothroyd RA, Chen HJ (2008). The psychometric properties of the Colorado Symptom Index. Admin Pol Ment Health.

[71] Trebble TM, Hansi N, Hydes T, Smith MA, Baker M (2010). Process mapping the patient journey through health care: an introduction. Brit Med J.

[72] Kaplan RS, Norton DP (2004). Strategy Mapping. Converting Intangible Assets into Tangible Outcomes.

[73] Enders C (2010). Applied missing data analysis.

[74] Wilcox R. Introduction to robust estimation and hypothesis testing. San Diego: Academic Press (Third edition). 2012.

[75] Baldwin SA, Murray DM, Shadish WR (2005). Empirically supported treatments or Type-I errors?: Problems with the analysis of data from group-administered treatments. J Consult Clinical Psych.

[76] Jaccard J, Bo A (2018). Prevention Science and Child/Youth Development: Randomized Explanatory Trials for Integrating Theory, Method, and Analysis in Program Evaluation. Journal of the Society of Social Work and Research.

[77] Glaser BG (1965). The constant comparative method of qualitative analysis. Soc Probl.

